# The Top 100 Most Cited Articles in Reconstructive Microsurgery: Identifying Evidence Gaps and Research Trends for Future Investigation: A Bibliometric and Visualization Analysis

**DOI:** 10.1055/a-2885-8984

**Published:** 2026-07-23

**Authors:** Hatan Mortada, Mustafa Qais Al-Khafaji, Zainalabden E. Jefri, Kian Daneshi, Abdulelah Alwadai, Chihiro Matsui, Feras Alshomer

**Affiliations:** 1 Division of Plastic Surgery, Department of Surgery 534884 King Saud University Medical CityRiyadh, Riyadh ProvinceSaudi Arabia; 2 Department of Plastic Surgery and Burn 149994 King Saud Medical CityRiyadh, Riyadh ProvinceSaudi Arabia; 3 Department of Plastic Surgery 6107 Norfolk and Norwich University Hospital NHS TrustNorwich, EnglandUnited Kingdom; 4 College of Medicine 37848 Faculty of Medicine, King Abdulaziz UniversityJeddah, Makkah ProvinceSaudi Arabia; 5 School of Population Health and Medicine 685422 Institute for Sustainable Food, The University of SheffieldSheffield, EnglandUnited Kingdom; 6 Department of BioengineeringImperial College LondonLondonUnited Kingdom; 7 Department of Plastic Surgery and Burn Unit 48103 Aseer Central HospitalAbha, Aseer ProvinceSaudi Arabia; 8 Department of Plastic and Reconstructive Surgery 73362 Juntendo University School of MedicineBunkyo, TokyoJapan; 9 Department of Plastic and Reconstructive Surgery 200682 National Center for Global Health and MedicineTokyoJapan; 10 Department of Plastic and Reconstructive Surgery 48149 King Saud bin Abdulaziz University for Health SciencesRiyadh, Riyadh ProvinceSaudi Arabia

**Keywords:** microsurgery, free tissue flaps, bibliometric analysis, reconstructive surgical procedures, lymphedema

## Abstract

**Background:**

Reconstructive microsurgery restores form and function after trauma, oncological resection, or congenital deformities. This study provides a bibliometric analysis of the most cited articles, identifies evidence gaps, and proposes future research priorities.

**Methods:**

The top 100 most cited articles were retrieved from Scopus on January 1, 2025, and analyzed for publication trends, authorship, themes, and impact metrics using VOSviewer and R Bibliometrix.

**Results:**

From 57,925 articles, the top 100 were selected (mean citations: 251.99 ± 207.78), predominantly in Q1 journals. Head and neck reconstruction dominated (49.5%). Hidalgo D.A. was the most frequent author, and the United States contributed nearly half of all articles. Most provided Level III–IV evidence (38.14% each); none met Level I criteria. Annual citation rates increased from 6.87 (1970s) to 16.32 (2010s). Four evolutionary phases were identified: Technical feasibility, clinical refinement, evidence-based optimization, and emerging interdisciplinary standardization.

**Conclusions:**

Three critical findings emerged: (1) No Level I studies and only 7.22% Level II evidence; (2) geographic concentration, with nearly half of contributions from the United States; and (3) a thematic shift from anatomical descriptions toward outcomes research. These findings provide a roadmap for advancing the field.

## Introduction

Reconstructive microsurgery is a specialized surgical technique in which surgeons repair or reconstruct lost or damaged tissues using operating microscopes and precision instruments to operate on delicate structures, such as blood vessels and nerves, that may be just a few millimeters in diameter. 1 It often involves transferring tissue from one part of the body to another, a technique commonly used in head and neck reconstructions following tumor ablation. 2 This discipline plays a critical role in restoring form and function after trauma, chronic wounds, oncological resections, and congenital deformities, 3 4 5 and is relevant to multiple fields, including plastic surgery, orthopaedic surgery, neurosurgery, and otolaryngology.

The field has evolved considerably, from ancient principles established by Sushruta to state-of-the-art innovations, such as the neurotization techniques described by Chang, which now allow restoration of sensation in reconstructed areas like the breast. 6 7 These advancements have expanded the scope and outcomes of procedures, including breast, head and neck, and extremity reconstructions.

Bibliometric analysis is an effective tool for quantitatively analyzing publication data to reveal patterns in research productivity, collaboration, and thematic development. 8 9 This approach maps the current state of knowledge, identifies gaps, and highlights future research directions. 8 10 However, comprehensive bibliometric analyses of reconstructive microsurgery have been limited. Previous studies have explored influential publications across various surgical domains, including breast microsurgery publication trends, 11 autologous breast reconstruction, 12 aesthetic plastic surgery, 13 brachial plexus injury, 14 hand microsurgery, 15 global microsurgery trends, 16 and high-impact microsurgery articles, 17 but most focused on a single subspecialty, were restricted to narrow timeframes, or relied primarily on descriptive cataloging without analyzing evidence quality, temporal citation dynamics, or translating findings into actionable recommendations. To date, no bibliometric study in reconstructive microsurgery has combined a comprehensive scope across all anatomical sites and five decades with an interpretive analytical framework that evaluates evidence-level trends, time-dependent citation behavior, open-access impact, and thematic evolution to generate a data-driven research agenda for the field. This study addresses these gaps through a novel interpretive bibliometric approach that moves beyond descriptive listing to answer critical questions: Where are the evidence gaps? Which research areas are oversaturated versus neglected? How has the quality of evidence evolved? By systematically analyzing evidence levels, geographic distribution, thematic evolution, time-dependent citation dynamics, and publication accessibility across the top 100 most cited studies, we aim to transform raw citation data into a strategic research agenda for reconstructive microsurgery.

## Methods

### Databases and Search Strategy

On January 1, 2025, a Scopus database search was conducted to identify the most cited articles in reconstructive microsurgery. Scopus was selected as the primary database for this analysis because it is the largest abstract and citation database of peer-reviewed literature, encompassing over 27,000 active titles across all disciplines. Compared to Web of Science, Scopus provides broader journal coverage, particularly for non-U.S. and non-European publications, and includes a wider range of surgical and biomedical journals relevant to reconstructive microsurgery. Unlike PubMed, which does not provide standardized citation counts or bibliometric export functionality, Scopus offers comprehensive citation metrics, author-level data, and institutional affiliation details essential for bibliometric analysis. Additionally, Scopus has been widely used and validated as the primary database in previous bibliometric studies across surgical specialties, including plastic surgery, microsurgery, and reconstructive surgery, supporting its suitability for this analysis. The resulting dataset represents the most cited articles retrievable through the specified Scopus search strategy and should not be interpreted as an exhaustive inventory of all historically influential publications in reconstructive microsurgery.

The search strategy employed the following terms in the title, abstract, and keyword fields: “reconstructive microsurgery,” “reconstructive surgery,” “microsurgery,” “microvascular surgery,” “free flap,” “vascularized bone graft,” and “tissue transfer.” Boolean operators (OR) were used to combine search terms, and the search was limited to articles published in English in peer-reviewed journals. No date restrictions were applied during the initial search. The search retrieved a total of 57,925 articles.

**Stage 1, Initial retrieval and ranking** : A total of 57,925 articles were retrieved from the Scopus database. All articles were ranked by total citation count in descending order, and the top 1,000 most cited articles were exported for further screening.

**Stage 2, Relevance screening** : Titles and abstracts of the top 1,000 articles were screened to identify studies relevant to reconstructive microsurgery, yielding 350 potentially eligible articles. The remaining 650 articles addressed non-reconstructive microsurgical topics and were excluded at this stage.

**Stage 3, Eligibility assessment** : The 350 articles were further assessed for eligibility, resulting in the exclusion of 250 articles: 200 focused on other surgical procedures without a primary reconstructive microsurgical component, and 50 were irrelevant to the topic. The remaining 100 articles were included in the final analysis ( Fig. 1 ).

**Fig. 1:**
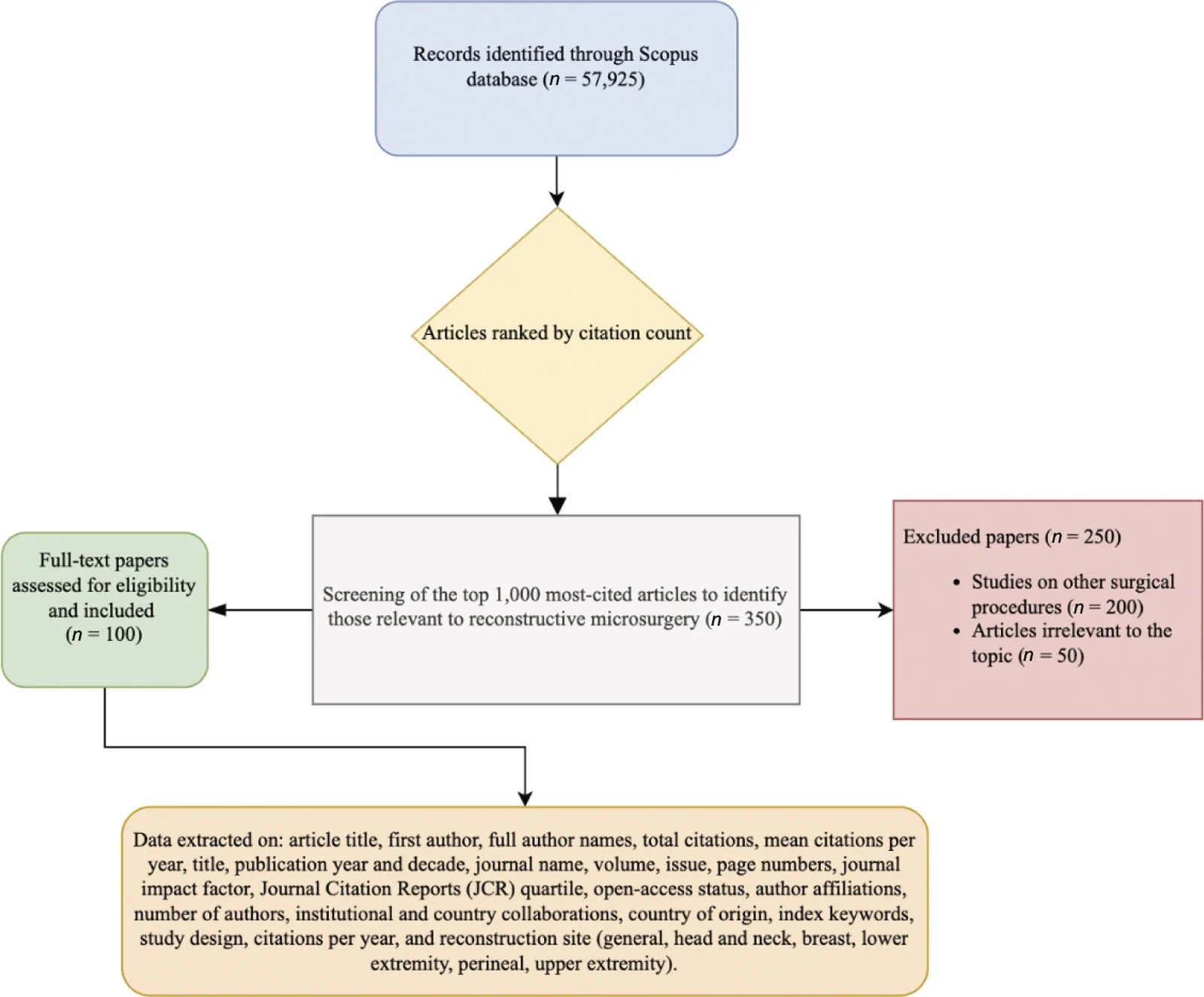
Flowchart outlining the study methodology. A three-stage screening process was applied: Initial retrieval and ranking of 57,925 articles from Scopus, relevance screening of the top 1,000 most cited articles by two independent reviewers, and final selection and ranking of the top 100 articles for bibliometric analysis.

#### Inclusion Criteria

(1) Original research articles, systematic reviews, narrative reviews, or meta-analyses published in peer-reviewed journals; (2) studies specifically addressing reconstructive microsurgical techniques, including any form of free flap surgery, vascularized tissue transfer, or microvascular reconstruction; (3) studies involving human subjects who underwent flap surgery or cadaveric dissections related to flap anatomy and techniques; (4) articles published in the English language; and (5) articles with at least one citation from another peer-reviewed source.

#### Exclusion Criteria

(1) Conference proceedings, abstracts, posters, editorials, letters to the editor, book chapters, or other non-peer-reviewed sources; (2) articles published in languages other than English; (3) articles not primarily addressing microsurgical or flap-related topics; (4) articles with zero citations; and (5) duplicate records across search results.

### Study Selection

This review included studies published in English between 1973 and 2018 that specifically focused on various types of flaps. Eligible studies involved human subjects who underwent any form of flap surgery or cadaveric dissections related to flap anatomy and techniques. Only original research articles and systematic or narrative reviews published in peer-reviewed journals, with citations in other publications, were considered for inclusion.

We limited the final analysis to the top 100 most cited articles because this threshold is commonly used in bibliometric studies to permit focused evaluation of the most influential literature while maintaining feasibility for detailed manual review. Restricting the sample to 100 articles also allowed article-level assessment of evidence classification, thematic evolution, and interpretive trends that would not be practical in a substantially larger dataset. A review of articles immediately beyond the cutoff (ranks 101–105) confirmed that the principal findings remain stable across marginally different thresholds.

### Metrics Analyzed

Data were systematically extracted from the included studies using a standardized Google Sheets (Google, Mountain View, CA, United States) extraction form. The first author (H.H.M.) supervised data management to ensure consistency and accuracy. For each article, the following variables were extracted: Article title, first author, full author names, total citations, mean citations per year, title, publication year and decade, journal name, volume, issue, page numbers, journal impact factor, Journal Citation Reports quartile, open-access status, author affiliations, number of authors, institutional and country collaborations, country of origin, index keywords, study design, citations per year, and reconstruction site (general, head and neck, breast, lower extremity, perineal, upper extremity). First authorship was selected as the primary metric for identifying the most prolific contributors, as it is the most consistently indexed variable in bibliometric databases and reflects direct leadership of study design and manuscript preparation. Data extraction was performed independently by two reviewers using a standardized extraction form, with discrepancies resolved by consensus.

Flap types were categorized as follows: Perforator flaps (e.g., deep inferior epigastric perforator [DIEP], anterolateral thigh [ALT], superior gluteal artery perforator [SGAP]), musculo/musculocutaneous flaps (e.g., transverse rectus abdominis myocutaneous [TRAM], gracilis), osseous/osteocutaneous flaps (e.g., fibular, iliac crest, scapular), fasciocutaneous flaps (e.g., radial forearm), fascial flaps (e.g., temporoparietal, parascapular), cutaneous flaps (e.g., groin flap), and visceral flaps (e.g., omental flap). Additionally, articles were subcategorized by main subject, keywords, H-index contribution, and level of evidence based on both standard classification (Levels I–V) and the Oxford Centre for Evidence-Based Medicine (OCEBM) criteria. 18 Funding sources were also recorded. This process identified the top 100 most cited articles in microsurgery for bibliometric analysis, providing insight into research impact, citation trends, and methodological quality in the field.

### Analytical Tools and Visualization Methods

Bibliometric analysis was conducted using the R Bibliometrix package and Shiny for data visualization, 9 while VOSviewer was employed for network mapping and cluster analysis. 19 Microsoft Excel was used for frequency analysis and descriptive statistics. Key bibliometric indicators, including author contributions, keyword occurrences, and research trends, were systematically extracted and summarized. Authors were ranked by publication count, keywords by frequency, and research topics were mapped over time to identify thematic evolution. Co-authorship networks were analyzed to assess collaboration patterns, and VOSviewer was used to construct keyword co-occurrence networks to identify thematic clusters and interconnections. Citation networks and institutional collaborations were also explored to assess the influence of key studies and research institutions in the field.

Time-dependent citation trends were assessed by calculating the annual citation rate (total citations divided by article age as of January 2025) and stratifying results by publication decade and article maturity. A focused subanalysis was performed on articles published within the most recent decade (2010–2018) to characterize contemporary research priorities and emerging thematic directions, comparing these with pre-2010 articles with respect to research themes, study designs, evidence levels, geographic distribution, and citation dynamics.

This manuscript was prepared in accordance with the Guideline for Reporting Bibliometric Reviews of the Biomedical Literature (BIBLIO). 20

## Results

### Article Selection and Citation Analysis

A total of 57,925 articles were initially retrieved from the Scopus database using predefined keywords. After ranking by citation count, the top 1,000 most cited studies were screened for relevance, identifying 350 articles related to reconstructive microsurgery. Of these, 250 were excluded (200 for addressing other surgical procedures and 50 for being irrelevant), yielding 100 articles for final analysis ( Fig. 1 and Table 1 ). The mean citation count was 251.99 ± 207.78 (range: 139–1,397; median: 182). The top quartile included articles with 273 or more citations, and only four articles exceeded 500 citations.

**Table 1:** List of the top 100 most cited papers in reconstructive microsurgery

Rank	First author	Title	Subject area	Year	Cited by	Mean citations/year	Volume	Issue	Page start	Page end
1	Hidalgo D.A.	Fibula free flap: A new method of mandible reconstruction.	Mandible/orofacial reconstruction	1989	1,397	38.81	84	1	71	79
2	Taylor G.I.	The free vascularized bone graft: A clinical extension of microvascular techniques.	Vascularized bone graft	1975	1,313	26.26	55	5	533	544
3	Song Y.G.	The free thigh flap: A new free flap concept based on the septocutaneous artery	Novel flap technique	1984	1,201	29.29	37	–	149	159
4	Khouri R.K.	A prospective study of microvascular free-flap surgery and outcome	Free flap outcomes (multisite)	1998	620	22.96	102	3	711	720
5	Bui D.T.	Free flap reexploration: Indications, treatment, and outcomes in 1,193 free flaps.	Free flap salvage	2007	487	27.06	119	7	2092	2100
6	Cordeiro P.G.	Reconstruction of the mandible with osseous free flaps: A 10-year experience with 150 consecutive patients.	Mandible/orofacial reconstruction	1999	433	16.65	104	5	1314	1320
7	Urken M.L.	Microvascular free flaps in head and neck reconstruction: Report of 200 cases and review of complications.	Head and neck free flap outcomes	1994	396	12.77	120	6	633	640
8	Kroll S.S.	Timing of pedicle thrombosis and flap loss after free-tissue transfer.	Free flap outcomes (multisite)	1996	392	13.52	98	7	1230	1233
9	Hidalgo D.A.	A review of 60 consecutive fibula free flap mandible reconstructions.	Mandible/orofacial reconstruction	1995	388	12.93	96	3	597	602
10	Taylor G.I.	The anatomy of several free flap donor sites.	Flap anatomy	1975	388	7.76	56	3	243	253
11	Khouri R.K.	Reconstruction of the lower extremity with microvascular free flaps: A 10-year experience with 304 consecutive cases.	Lower extremity free flap outcomes	1989	378	10.5	29	8	1086	1094
12	Jewer D.D.	Orofacial and mandibular reconstruction with the iliac crest free flap: A review of 60 cases and a new method of classification.	Mandible/orofacial reconstruction	1989	376	10.44	84	3	391	403
13	Chen K-T.	Timing of presentation of the first signs of vascular compromise dictates the salvage outcome of free flap transfers.	Free flap salvage	2007	376	20.89	120	1	187	195
14	May J.W., Jr	The no-reflow phenomenon in experimental free flaps.	Ischemia/reflow phenomenon	1978	372	7.91	61	2	256	267
15	Dort J.C.	Optimal perioperative care in major head and neck cancer surgery with free flap reconstruction: A consensus review and recommendations from the enhanced recovery after surgery society.	Perioperative care/guidelines	2017	367	45.88	143	3	291	303
16	Schusterman M.A.	A single center's experience with 308 free flaps for repair of head and neck cancer defects.	Head and neck free flap outcomes	1994	340	10.97	93	3	472	478
17	Urken M.L.	Oromandibular reconstruction using microvascular composite free flaps: report of 71 cases and a new classification scheme for bony, soft-tissue, and neurologic defects.	Mandible/orofacial reconstruction	1991	326	9.59	117	7	733	744
18	Khouri R.K.	Avoiding free flap failure.	Free flap complication prevention	1992	322	9.76	19	4	773	781
19	Wei F-C.	Free-style free flaps.	Novel flap technique	2004	316	15.05	114	4	910	916
20	Allen R.J.	Superior gluteal artery perforator free flap for breast reconstruction.	Breast reconstruction	1995	311	10.37	95	7	1207	1212
21	Haughey B.H.	Free flap reconstruction of the head and neck: Analysis of 241 cases.	Head and neck free flap outcomes	2001	305	12.71	125	1	10	17
22	Hidalgo D.A.	Free-flap mandibular reconstruction: A 10-year follow-up study.	Mandible/orofacial reconstruction	2002	300	13.04	110	2	438	449
23	Kroll S.S.	Choice of flap and incidence of free flap success.	Free flap outcomes (multisite)	1996	300	10.34	98	3	459	463
24	Nakatsuka T.	Analytic review of 2372 free flap transfers for head and neck reconstruction following cancer resection.	Head and neck free flap outcomes	2003	290	13.18	19	6	363	368
25	Disa J.J.	Efficacy of conventional monitoring techniques in free tissue transfer: An 11-year experience in 750 consecutive cases.	Flap monitoring	1999	276	10.62	104	1	97	101
26	Serletti J.M.	Factors affecting outcome in free-tissue transfer in the elderly.	Free flap outcomes (multisite)	2000	273	10.92	106	1	66	70
27	Hidalgo D.A.	A review of 716 consecutive free flaps for oncologic surgical defects: Refinement in donor-site selection and technique.	Free flap outcomes (multisite)	1998	271	10.04	102	3	722	732
28	Wong C-H.	Microsurgical free flap in head and neck reconstruction.	Head and neck free flap outcomes	2010	269	17.93	32	9	1236	1245
29	Song R.	The upper arm free flap.	Novel flap technique	1982	266	6.19	9	1	27	35
30	Blondeel P.N.	Refinements in free flap breast reconstruction: The free bilateral deep inferior epigastric perforator flap anastomosed to the internal mammary artery.	Breast reconstruction	1994	260	8.39	47	7	495	501
31	Brent B.	Secondary ear reconstruction with cartilage grafts covered by axial, random, and free flaps of temporoparietal fascia.	Ear reconstruction	1983	255	6.07	72	2	141	151
32	Nassif T.M.	The parascapular flap: A new cutaneous microsurgical free flap.	Novel flap technique	1982	246	5.72	69	4	591	600
33	Clark J.R.	Predictors of morbidity following free flap reconstruction for cancer of the head and neck.	Head and neck complication analysis	2007	229	12.72	29	12	1090	1101
34	Brent B.	Experience with the temporoparietal fascial free flap.	Ear reconstruction	1985	228	5.7	76	2	177	188
35	Hidalgo D.A.	The role of emergent exploration in free-tissue transfer: A review of 150 consecutive cases.	Emergent flap exploration	1990	223	6.37	86	3	492	498
36	Godina M.	Preferential use of end-to-side arterial anastomoses in free flap transfers.	Microsurgical anastomosis technique	1979	220	4.78	64	5	673	682
37	Brown J.S.	Deep circumflex iliac artery free flap with internal oblique muscle as a new method of immediate reconstruction of maxillectomy defect.	Maxillectomy/midface reconstruction	1996	213	7.34	18	5	412	421
38	Cavadas P.C.	The medial sural artery perforator free flap.	Novel flap technique	2001	209	8.71	108	5	1609	1615
39	Hidalgo D.A.	Aesthetic improvements in free-flap mandible reconstruction	Mandible/orofacial reconstruction	1991	204	6	88	4	574	585
40	Campisi C.	Microsurgery for lymphedema: Clinical research and long-term results.	Lymphedema microsurgery	2010	202	13.47	30	4	256	260
41	Smit J.M.	Advancements in free flap monitoring in the last decade: A critical review.	Flap monitoring	2010	202	13.47	125	1	177	185
42	Hanasono M.M.	A prospective study of donor-site morbidity after anterolateral thigh fasciocutaneous and myocutaneous free flap harvest in 220 patients.	Donor site morbidity	2010	199	13.27	125	1	209	214
43	Wei F-C.	Double free flaps in reconstruction of extensive composite mandibular defects in head and neck cancer.	Mandible/orofacial reconstruction	1999	195	7.5	103	1	39	47
44	Urken M.L.	Composite free flaps in oromandibular reconstruction: Review of the literature.	Mandible/orofacial reconstruction	1991	193	5.68	117	7	724	732
45	Boyd J.B.	The free flap and plate in oromandibular reconstruction long-term review and indications.	Mandible/orofacial reconstruction	1995	190	6.33	95	6	1018	1028
46	Basta M.N.	Operative treatment of peripheral lymphedema: A systematic meta-analysis of the efficacy and safety of lymphovenous microsurgery and tissue transplantation.	Lymphedema microsurgery	2014	185	16.82	133	4	905	913
47	Yu P.	Analysis of 49 cases of flap compromise in 1310 free flaps for head and neck reconstruction.	Free flap salvage	2009	184	11.5	31	1	45	51
48	Lovie M.J.	The ulnar artery forearm free flap.	Novel flap technique	1984	184	4.49	37	4	486	492
49	Harashina T.	Analysis of 200 free flaps.	Free flap outcomes (multisite)	1988	183	4.95	41	1	33	36
50	Bozikov K.	Factors predicting free flap complications in head and neck reconstruction.	Head and neck complication analysis	2006	182	9.58	59	7	737	742
51	Urken M.L.	The internal oblique-iliac crest osseomyocutaneous free flap in oromandibular reconstruction: Report of 20 cases.	Mandible/orofacial reconstruction	1989	182	5.06	115	3	339	349
52	Brown J.S.	Factors that influence the outcome of salvage in free tissue transfer.	Free flap salvage	2003	181	8.23	41	1	16	20
53	Rogers S.N.	Health-related quality of life after maxillectomy: A comparison between prosthetic obturation and free flap.	Quality of life assessment	2003	179	8.14	62	2	174	181
54	Kazuteru D.	Free vascularized bone graft for nonunion of the scaphoid.	Scaphoid reconstruction	2000	178	7.12	25	3	507	519
55	Patel R.S.	Clinicopathologic and therapeutic risk factors for perioperative complications and prolonged hospital stay in free flap reconstruction of the head and neck.	Head and neck complication analysis	2010	172	11.47	32	10	1345	1353
56	Wei F-C.	Complications after reconstruction by plate and soft-tissue free flap in composite mandibular defects and secondary salvage reconstruction with osteocutaneous flap.	Mandible/orofacial reconstruction	2003	172	7.82	112	1	37	42
57	Hölzle F.	Free flap monitoring using simultaneous non-invasive laser Doppler flowmetry and tissue spectrophotometry.	Flap monitoring	2006	171	9	34	1	25	33
58	Bianchi B.	Free flaps: Outcomes and complications in head and neck reconstructions.	Head and neck free flap outcomes	2009	168	10.5	37	8	438	442
59	Pestana I.A.	Early experience with fluorescent angiography in free-tissue transfer reconstruction.	Flap monitoring	2009	167	10.44	123	4	1239	1244
60	Taylor G.I.	The current status of free vascularized bone grafts.	Vascularized bone graft	1983	167	3.98	10	1	185	209
61	Coleman J.J.	The bipedicled osteocutaneous scapula flap: A new subscapular system free flap.	Novel flap technique	1991	166	4.88	87	4	682	692
62	Weiland A.J.	The efficacy of free tissue transfer in the treatment of osteomyelitis.	Osteomyelitis reconstruction	1984	164	4	66	2	181	93
63	Baek S.M.	Two new cutaneous free flaps: The medial and lateral thigh flaps.	Novel flap technique	1983	164	3.9	71	3	354	65
64	Yuen J.C.	Monitoring free flaps using the laser Doppler flowmeter: Five-year experience.	Flap monitoring	2000	163	6.52	105	1	55	61
65	Lister G.	Emergency free flaps to the upper extremity.	Upper extremity reconstruction	1988	162	4.38	13	1	22	28
66	Harii K.	Successful clinical transfer of ten free flaps by microvascular anastomoses.	Free flap outcomes (multisite)	1974	162	3.18	53	3	259	70
67	Harii K.	Pharyngoesophageal reconstruction using a fabricated forearm free flap.	Pharyngoesophageal reconstruction	1985	161	4.03	75	4	463	476
68	Cho E.H.	Muscle versus fasciocutaneous free flaps in lower extremity traumatic reconstruction: A multicenter outcomes analysis.	Lower extremity free flap outcomes	2018	160	22.86	141	1	191	199
69	Novakovic D.	Salvage of failed free flaps used in head and neck reconstruction.	Free flap salvage	2009	160	10	1	Not applicable	article 33	Not applicable
70	Vyas R.M.	Risk factors for abdominal donor-site morbidity in free flap breast reconstruction.	Donor site morbidity	2008	158	9.29	121	5	1519	1526
71	Disa J.J.	Mandible reconstruction with microvascular surgery.	Mandible/orofacial reconstruction	2000	158	6.32	19	3	226	234
72	Boyd J.	Recipient vessels in head and neck microsurgery: Radiation effect and vessel access.	Head and neck free flap outcomes	1993	156	4.88	92	4	628	632
73	Evans G.R.D.	The radial forearm free flap for head and neck reconstruction: A review.	Head and neck reconstruction review	1994	156	5.03	168	5	446	450
74	Blackwell K.E.	Unsurpassed reliability of free flaps for head and neck reconstruction.	Head and neck free flap outcomes	1999	154	5.92	125	3	295	299
75	Futran N.D.	Midface reconstruction with the fibula free flap.	Maxillectomy/midface reconstruction	2002	153	6.65	128	2	161	166
76	Shaw W.W.	Breast reconstruction by superior gluteal microvascular free flaps without silicone implants.	Breast reconstruction	1983	153	3.64	72	4	490	501
77	Mathoulin C.	Vascularized bone graft from the palmar carpal artery for treatment of scaphoid nonunion.	Scaphoid reconstruction	1998	152	5.63	23	3	318	323
78	Jones D.B.	Treatment of scaphoid waist nonunions with an avascular proximal pole and carpal collapse. A comparison of two vascularized bone grafts.	Scaphoid reconstruction	2008	151	8.88	90	12	2612	2625
79	Morrison W.A.	The instep of the foot as a fasciocutaneous island and as a free flap for heel defects.	Lower extremity reconstruction	1983	151	3.6	72	1	56	65
80	Acland R.D.	The saphenous neurovascular free flap.	Novel flap technique	1981	149	3.39	67	6	763	774
81	Takushima A.	Mandibular reconstruction using microvascular free flaps: A statistical analysis of 178 cases.	Mandible/orofacial reconstruction	2001	149	6.21	108	6	1556	1563
82	Khouri R.K.	Prefabrication of composite free flaps through staged microvascular transfer: An experimental and clinical study.	Flap prefabrication	1991	148	4.35	87	1	108	115
83	Moreno M.A.	Microvascular free flap reconstruction versus palatal obturation for maxillectomy defects.	Maxillectomy/midface reconstruction	2010	147	9.8	32	7	860	867
84	Kruse A.L.D.	Factors influencing survival of free-flap in reconstruction for cancer of the head and neck: A literature review.	Head and neck free flap outcomes	2010	147	9.8	30	3	242	248
85	Hill H.L.	The tensor fascia lata myocutaneous free flap.	Novel flap technique	1978	147	3.13	61	4	517	522
86	Anthony J.P.	Donor leg morbidity and function after fibula free flap mandible reconstruction.	Donor-site morbidity	1995	146	4.87	96	1	146	152
87	Taylor G.I.	The free flap: Composite tissue transfer by vascular anastomosis.	Microsurgical technique (composite tissue transfer)	1973	146	2.81	43	1	1	3
88	Sadove R.C.	Reconstruction of the lower lip and chin with the composite radial forearm-palmaris longus free flap.	Nasal/lip reconstruction	1991	146	4.29	88	2	209	214
89	Pohlenz P.	Microvascular free flaps in head and neck surgery: Complications and outcome of 1000 flaps.	Head and neck free flap outcomes	2012	144	11.08	41	6	739	743
90	Triana R.J., Jr.	Microvascular free flap reconstructive options in patients with partial and total maxillectomy defects.	Maxillectomy/midface reconstruction	2000	144	5.76	2	2	91	101
91	Wei F-C.	The outcome of failed free flaps in head and neck and extremity reconstruction: What is next in the reconstructive ladder?	Salvage/reconstructive ladder	2001	142	5.92	108	5	1154	1160
92	Weiland A.J.	Free vascularized bone grafts in surgery of the upper extremity	Vascularized bone graft	1979	142	3.09	4	2	129	144
93	Boyer M.I.	Scaphoid nonunion with avascular necrosis of the proximal pole: Treatment with a vascularized bone graft from the dorsum of the distal radius.	Scaphoid reconstruction	1998	142	5.26	29	9	686	690
94	Campisi C.	Microsurgical techniques for lymphedema treatment: Derivative lymphatic-venous microsurgery.	Lymphedema microsurgery	2004	142	6.76	28	6	609	613
95	Chang L.D.	Cigarette smoking, plastic surgery, and microsurgery.	Smoking and microsurgery	1996	142	4.9	12	7	467	474
96	Burget G.C.	Optimal use of microvascular free flaps, cartilage grafts, and a paramedian forehead flap for aesthetic reconstruction of the nose and adjacent facial units.	Nasal/lip reconstruction	2007	141	7.83	120	5	1171	1207
97	Momoh A.O.	A prospective cohort study of fibula free flap donor-site morbidity in 157 consecutive patients.	Donor site morbidity	2011	141	10.07	128	3	714	720
98	Yu P.	Comparison of clinical and functional outcomes and hospital costs following pharyngoesophageal reconstruction with the anterolateral thigh free flap versus the jejunal flap.	Pharyngoesophageal reconstruction	2006	140	7.37	117	3	968	974
99	Serafin D.	Comparison of free flaps with pedicled flaps for coverage of defects of the leg or foot.	Lower extremity free flap outcomes	1977	139	2.9	59	4	492	499
100	Smolka K.	Fibula free flap reconstruction of the mandible in cancer patients: Evaluation of a combined surgical and prosthodontic treatment concept.	Mandible/orofacial reconstruction	2008	139	8.18	44	6	571	581

### Time-Dependent Citation Trends

The annual citation rate (total citations divided by article age as of January 2025) was calculated for all 100 articles (mean: 9.76 ± 7.09; median: 8.03). A significant inverse correlation was observed between article age and annual citation rate (Spearman ρ = −0.610, *p* < 0.001), indicating that newer, highly cited articles accumulate citations at a faster pace. When stratified by publication decade, the mean annual citation rate increased progressively from 6.87 (1970s) to 8.32 (1980s), 8.56 (1990s), 10.07 (2000s), and 16.32 (2010s). When categorized by citation maturity, contemporary articles (≤15 years, *n* = 12) had a mean annual citation rate of 16.32, compared to 9.95 for established articles (15–30 years, *n* = 47) and 7.63 for foundational articles (>30 years, *n* = 41), consistent with the broader trend of increasing publication volume and digital accessibility in biomedical literature.

Despite this trend, five foundational articles published more than 35 years ago maintained annual citation rates exceeding 10: Hidalgo D.A. (1989; 38.81/year), Song Y.G. (1984; 29.29/year), Taylor G.I. (1975; 26.26/year), Khouri R.K. (1989; 10.50/year), and Jewer D.D. (1989; 10.44/year). These landmark contributions, introducing the fibula free flap, the free thigh flap concept, and the vascularized bone graft, continue to be actively cited decades after publication, suggesting enduring influence that transcends typical citation decay.

The temporal distribution of publications showed peak productivity during the 1990s and 2000s, which together contributed 60 of the 100 articles ( Fig. 2A ). The cumulative curve demonstrated steady growth from the 1970s through 2010, with a plateau thereafter reflecting the time required for recent articles to accumulate sufficient citations. Mean total citations decreased from 336.6 (1970s) to 194.6 (2010s), consistent with shorter citation windows for newer publications ( Fig. 2B ).

**Fig. 2:**
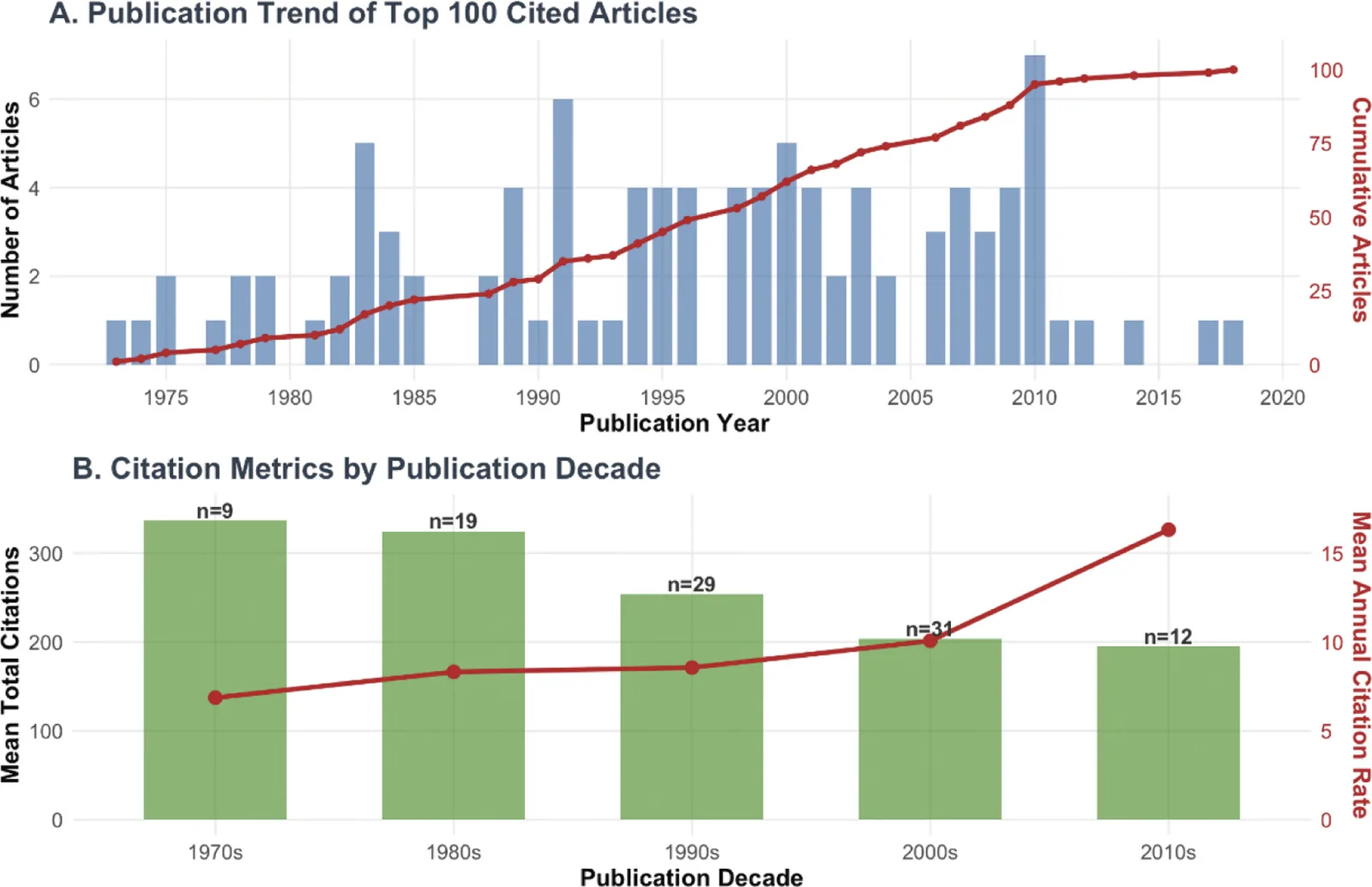
Citation trends of the 100 most cited articles in reconstructive microsurgery. ( **A** ) Number of articles entering the top 100 by publication year (blue bars) with cumulative article count (red line). ( **B** ) Mean total citations per article (green bars) and mean annual citation rate (red line) stratified by publication decade. The number of articles per decade is indicated above each bar ( *n* ).

### Publication Trends and Authors

The 100 most cited articles were published between 1973 and 2018 (median: 1996), with the highest output in the 2000s ( *n* = 31), followed by the 1990s ( *n* = 29), 1980s ( *n* = 19), 2010s ( *n* = 12), and 1970s ( *n* = 9). The distribution of citations over time is illustrated in Fig. 3 . Hidalgo D.A. was the most frequently listed author (eight articles), followed by Wei F-C. (six articles), Cordeiro P.G., Disa J.J., Miller M.J., Reece G.P., and Robb G.L. (five articles each), and Taylor G.I., Daniel R.K., and Gullane P.J. (four articles each; Fig. 4 ).

**Fig. 3:**
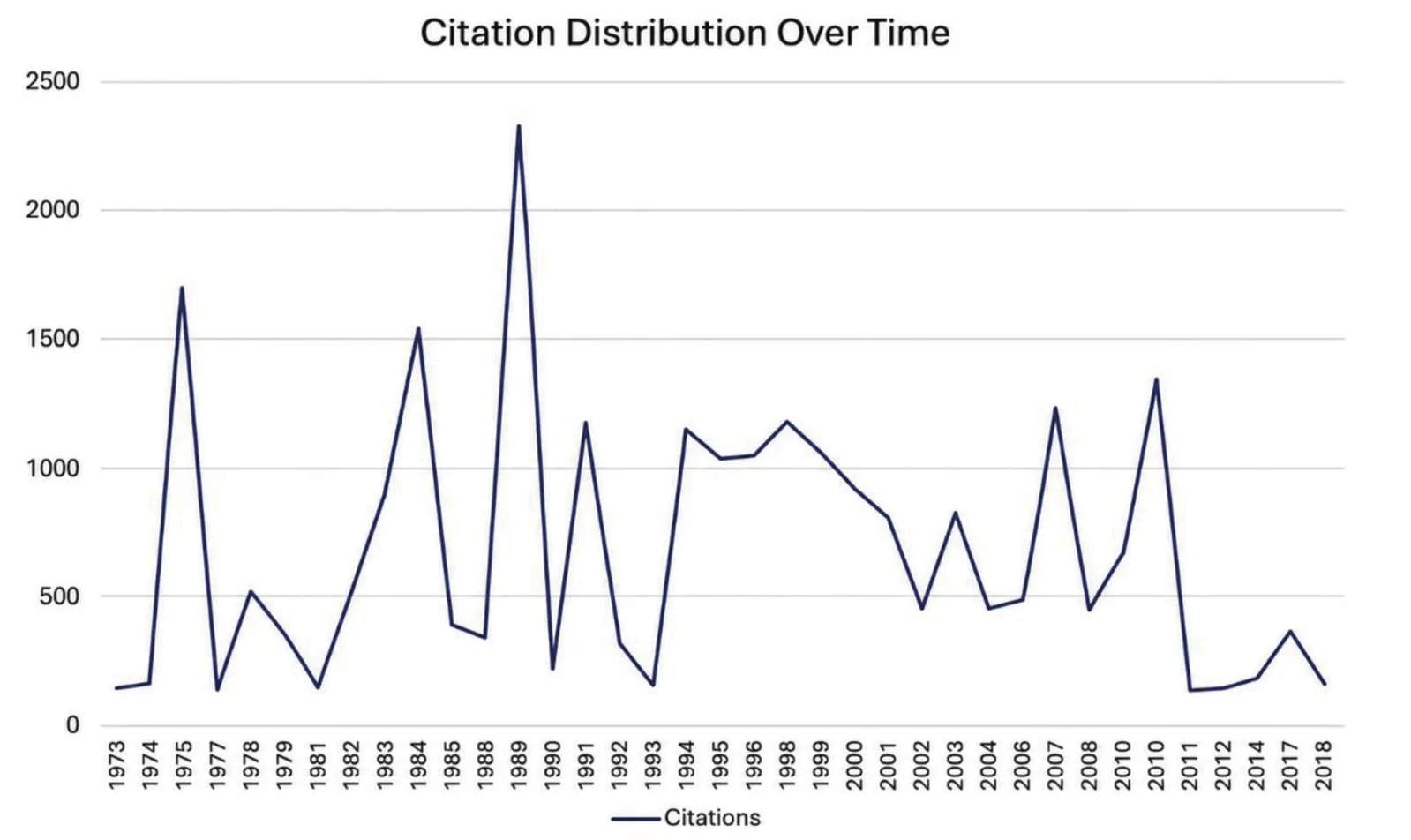
Citation distribution over time for the 100 most cited articles in reconstructive microsurgery.

**Fig. 4:**
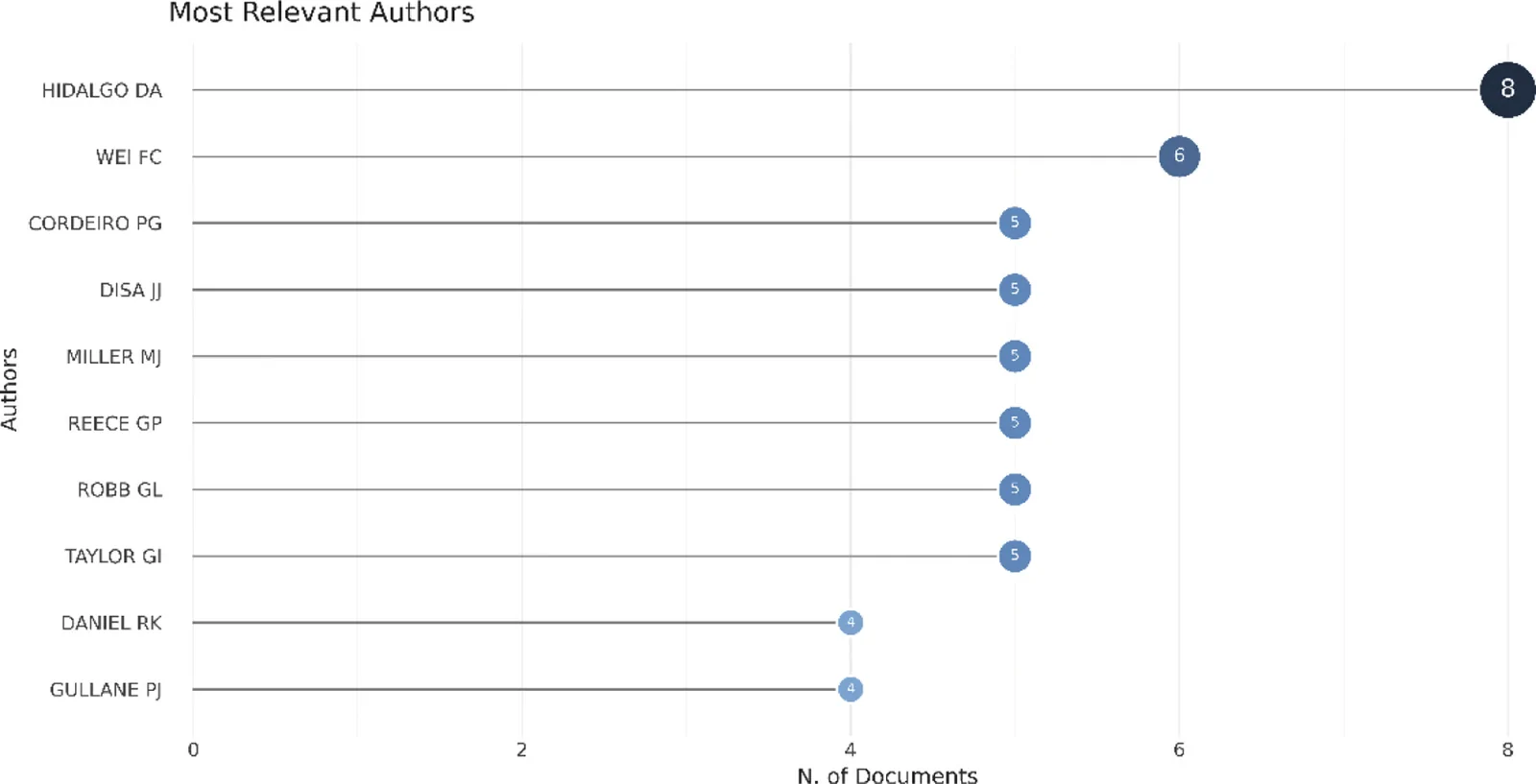
Most relevant authors in the 100 most cited articles in reconstructive microsurgery.

A comparison of authorship roles revealed that rankings remained largely stable regardless of metric ( Table 2 ). Hidalgo D.A. led across all positions (six first-authored, three senior-authored, eight total). However, senior authorship analysis revealed contributors not captured by first-author rankings: Daniel R.K. (four senior, zero first) served as senior investigator on landmark studies by Taylor G.I. and Weiland A.J.; Biller H.F. (three senior, zero first) supervised all three of Urken M.L.'s highly cited head and neck studies; and Shaw W.W. (three senior) mentored studies by Khouri R.K., among others. Cordeiro P.G. and Disa J.J. each appeared in five articles despite contributing only one and two first-authored articles, respectively, reflecting extensive collaborative involvement. Twelve articles were single-authored.

**Table 2:** Comparison of author rankings by authorship role

Author	First author ( *n* )	Last/Senior author ( *n* )	Any position ( *n* )
Hidalgo D.A.	6	3	8
Wei F-C.	4	2	7
Taylor G.I.	4	1	5
Khouri R.K.	4	1	4
Urken M.L.	4	1	4
Daniel R.K.	0	4	4
Shaw W.W.	1	3	4
Cordeiro P.G.	1	1	5
Disa J.J.	2	0	5
Biller H.F.	0	3	3

#### Journals, Impact Factor, and Open-Access Status

*Plastic and Reconstructive Surgery* contributed 54 articles, followed by *Head and Neck* (6 articles), *Archives of Otolaryngology–Head and Neck Surgery* (4 articles), *British Journal of Plastic Surgery* (4 articles), and *Clinics in Plastic Surgery* (3 articles; Fig. 5 ). The mean journal impact factor was 3.08 ± 1.06 (range: 1.0–6.1; median: 3.2). Among the 100 articles, 80 (82.47%) were published in Q1 journals, 15 (15.46%) in Q2, 1 (1.03%) in Q3, and 1 (1.03%) in Q4. Regarding open-access status, 50 articles (50%) were open-access, 12 (12%) were hybrid open-access, 37 (37%) were non-open-access, and 1 (1%) had unspecified status.

**Fig. 5:**
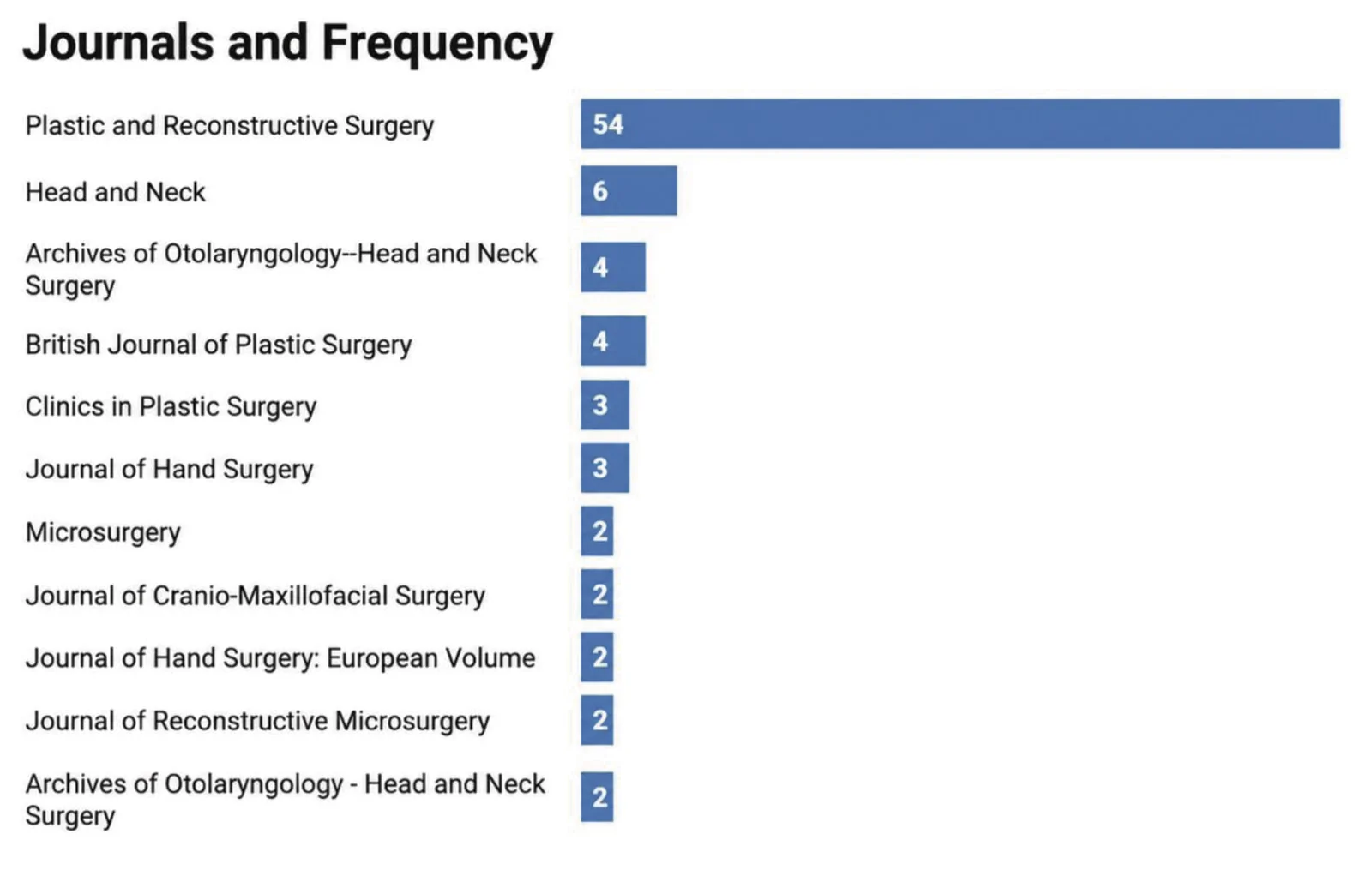
Distribution of the most cited articles in reconstructive microsurgery by journal.

Non-open-access articles had a higher mean citation count (331.87 ± 316.31) compared to open-access/hybrid articles (203.03 ± 54.50); however, this difference was driven by a small number of landmark historical articles with exceptionally high counts. The median citation count was higher for open-access/hybrid articles (183.5 vs. 159.0), and their lower variability (standard deviation [SD]: 54.50 vs. 316.31) suggests more consistent citation performance, supporting the broader adoption of open-access publishing in the field.

### Authorship, Geographic Distribution, and Institutional Contributions

The number of authors per article ranged from 1 to 11 (median: 4). H-index values of contributing authors ranged from 3 to 86 (mean: 34.85 ± 19.29). The United States contributed the most articles (49%), followed by Japan (6%), Canada (5%), Taiwan (5%), and Australia (4%; Fig. 6 ). Taiwan was initially undercounted due to Scopus misclassification of three Chang Gung Memorial Hospital (Taipei) articles as originating from China; after manual correction, Taiwan contributed 5% and China 2%. Regarding institutional collaboration, 46 articles (46%) were single-institution studies, 9 (9%) involved two institutions, and 2 (2%) involved three institutions. The University of Texas MD Anderson Cancer Center was the most frequently represented institution (3%), followed by Mount Sinai Medical Center (2%).

**Fig. 6:**
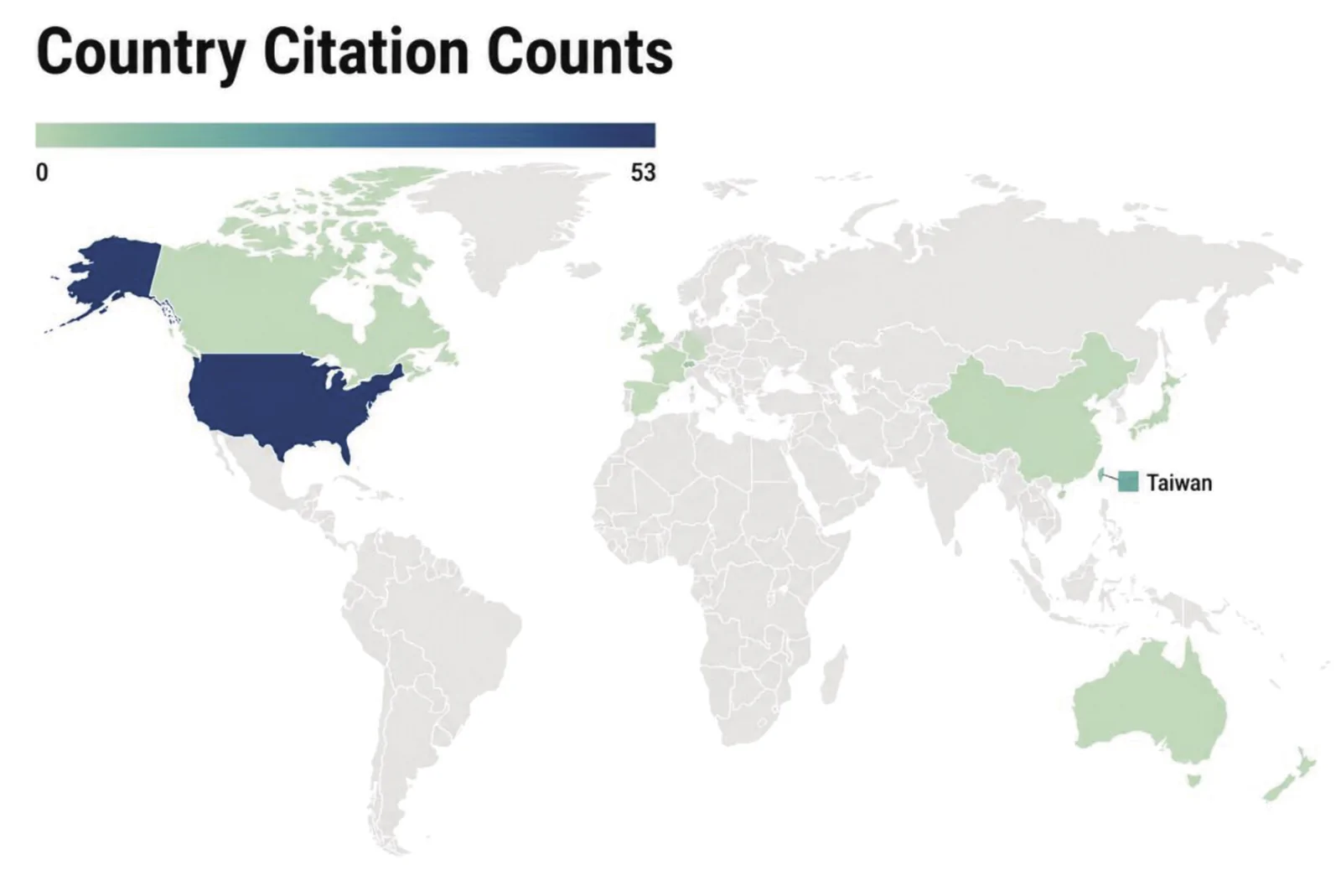
Distribution of the most cited articles in reconstructive microsurgery by country. Generated by the authors using the R Bibliometrix package based on Scopus data extracted on January 1, 2025.

### Study Design, Focus Areas, and Evidence Levels

Among the 100 articles, 19% were case series, 12% retrospective cohort studies, 9% retrospective studies, 8% retrospective reviews, 6% retrospective case series, 6% case reports, and 5% literature reviews. Head and neck reconstruction was the dominant focus (49.5%), followed by general/multisite reconstruction (26.9%), lower extremity (11.8%), upper extremity (7.5%), and breast reconstruction (4.3%). The primary research themes included free flap outcomes (23.3%), mandible and orofacial reconstruction (16.7%), novel flap techniques (13.3%), flap donor sites and morbidity (8.3%), and lymphedema microsurgical treatments (5.0%). The keyword co-occurrence network is illustrated in Figs. 7 and 8 .

**Fig. 7:**
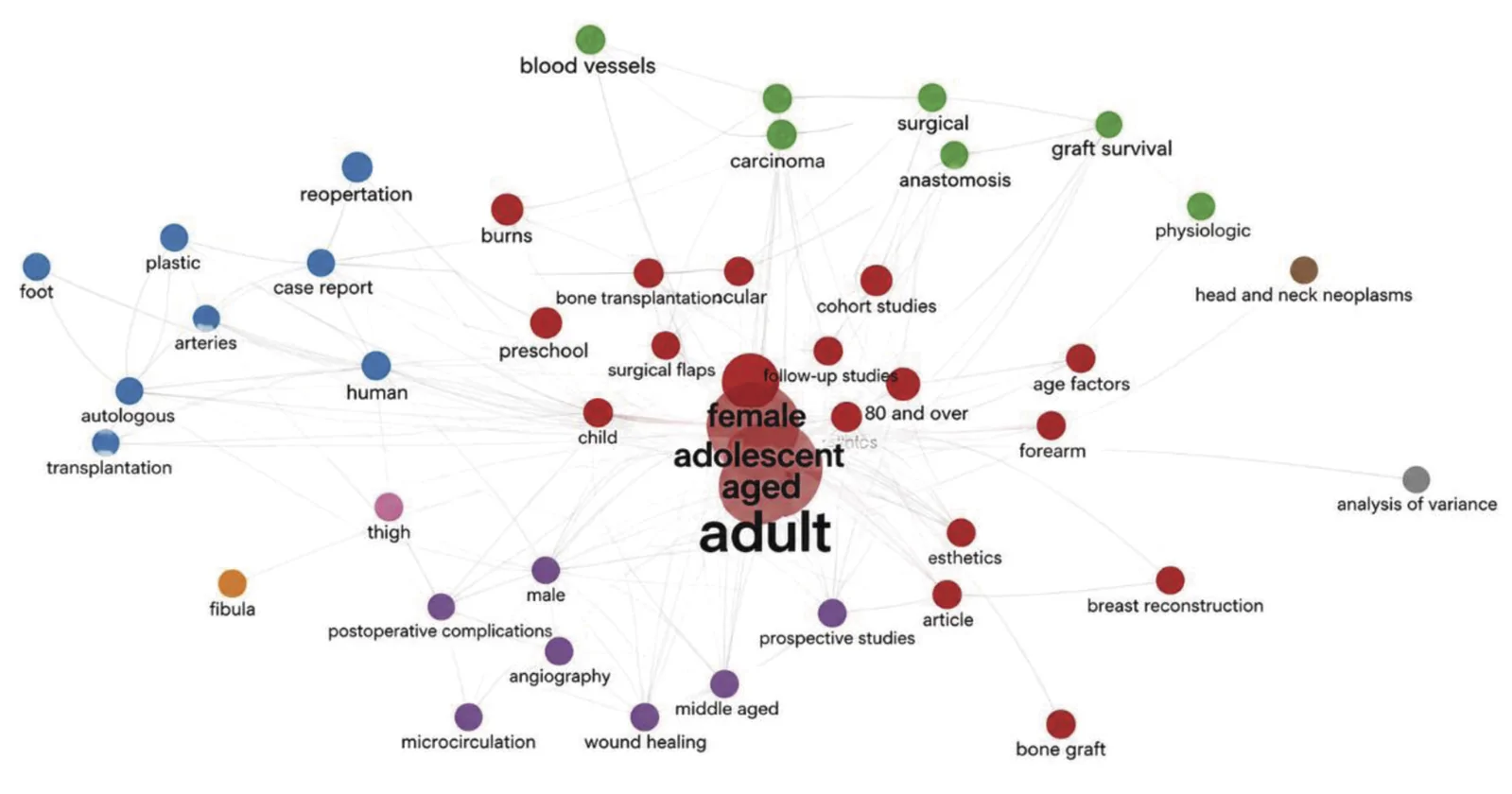
Keyword co-occurrence network in the 100 most cited articles in reconstructive microsurgery. Generated by the authors using VOSviewer based on keyword co-occurrence data from the included articles.

**Fig. 8:**
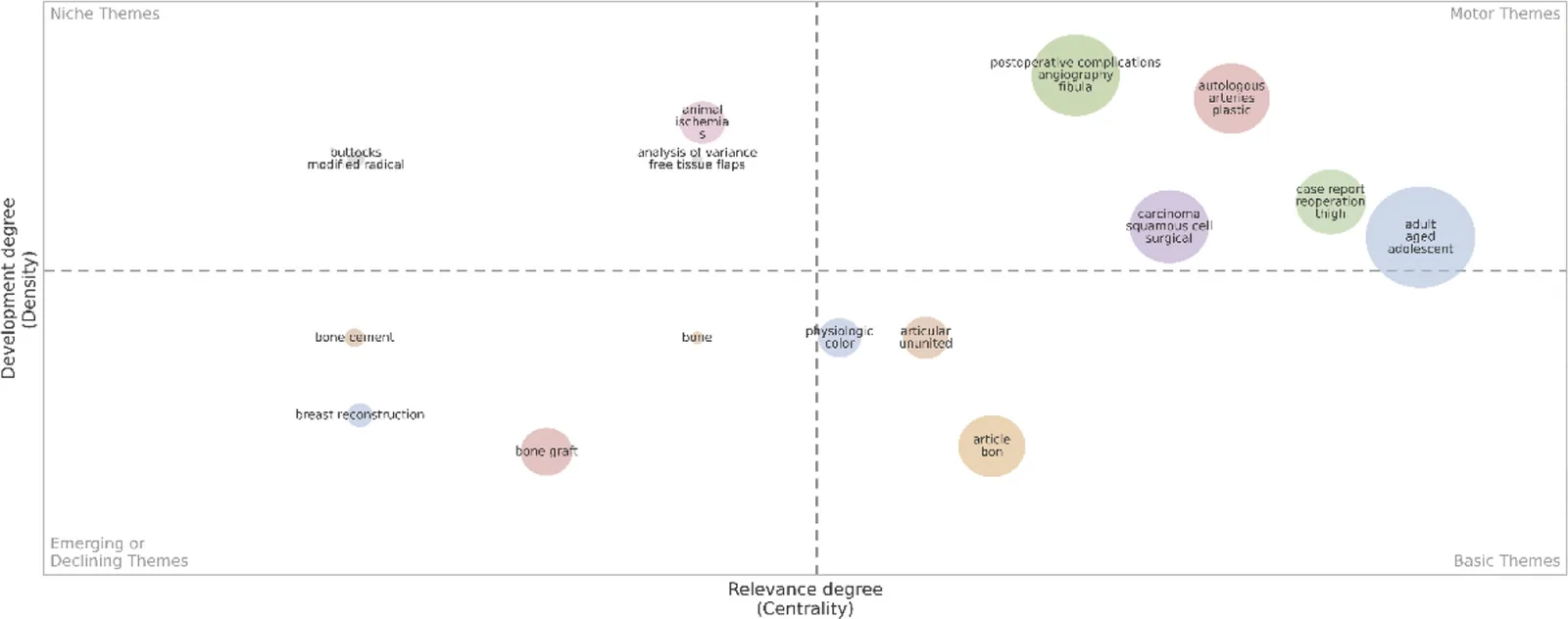
Thematic map of research topics in the 100 most cited articles in reconstructive microsurgery.

Among 97 articles with an assignable evidence level, the majority were Level III ( *n* = 37, 38.14%) and Level IV ( *n* = 37, 38.14%), followed by Level V ( *n* = 16, 16.49%) and Level II ( *n* = 7, 7.22%). No article met Level I criteria. Analysis across decades revealed gradual but incomplete methodological maturation: case reports and small case series declined from 62.5% (1970s–1980s) to 26.3% (2000s), while Level III evidence rose from absent in the 1970s to 61.3% in the 2000s. Level II evidence first appeared in the 1990s ( *n* = 3, 10.3%) but never exceeded 10.3% in any decade. The 2010s showed a paradoxical resurgence of Level V evidence (33.3%), driven by literature reviews and consensus guidelines, suggesting that the field's most influential recent contributions have taken the form of evidence synthesis rather than primary high-level research. Funding was unreported in the majority of studies, with 76 (76%) providing no information on financial support.

### Thematic Evolution and Paradigm Shifts

Analysis of the 100 most cited articles revealed four distinct evolutionary phases, each defined by characteristic research priorities, study designs, and landmark contributions.

- **Phase I, Technical feasibility (1973–1985;*n* = 22)** : The earliest articles focused on proving that free tissue transfer was technically achievable. Taylor G.I.'s 1973 description of composite tissue transfer (146 citations) and Harii K.'s 1974 report of 10 free flaps (162 citations) provided proof of concept, culminating with the introduction of workhorse donor sites: Song Y.G.'s free thigh flap (1984; 1,201 citations), which established the septocutaneous artery concept underpinning perforator flap surgery, and Taylor G.I.'s characterization of free flap donor site anatomy (1975; 388 citations). Evidence was predominantly Level IV (62.5%).
- **Phase II, Clinical refinement (1986–1999;*n* = 37)** : Hidalgo D.A.'s fibula free flap for mandibular reconstruction (1989; 1,397 citations, the most cited article in the field) transformed head and neck reconstruction. This era saw large single-institution case series (Schusterman M.A., 308 free flaps; Khouri R.K., 304 lower extremity cases) and the first systematic evaluations of complications and donor site morbidity. Level III evidence began to appear alongside the still-dominant Level IV studies.
- **Phase III, Evidence-based optimization (2000–2012;*n* = 38)** : This phase was characterized by multi-institutional reviews (Nakatsuka T., 2,372 free flap transfers), systematic analyses of complication predictors, and the introduction of monitoring technologies (laser Doppler, fluorescent angiography) and lymphedema microsurgery. Level III evidence peaked at 61.3%.
- **Phase IV, Standardization and integration (2013–2018;*n* = 3)** : Although limited by citation accumulation time, three articles signaled new directions: Dort J.C.'s enhanced recovery after surgery (ERAS) consensus guidelines (2017; 367 citations; 45.88 citations/year, the highest annual citation rate in the dataset), Basta M.N.'s systematic meta-analysis of lymphedema treatment (2014; 185 citations), and Cho E.H.'s multicenter outcomes analysis (2018; 160 citations). This phase included the only systematic review with meta-analysis in the top 100.

The prominence of Dort et al.'s ERAS consensus recommendations is particularly informative because it suggests that the field increasingly rewards publications that are scalable across centers and immediately translatable into perioperative practice. This differs from earlier citation patterns dominated by landmark anatomical and technical papers and may indicate a shift in how authority is produced within reconstructive microsurgery, from innovation at the level of operative technique toward influence at the level of systems of care.

Across all phases, the most enduringly cited articles were those that introduced novel conceptual frameworks (angiosome theory, septocutaneous artery concept), established new reconstructive standards, or provided large-scale outcome data. Articles describing incremental refinements generally achieved lower citation counts and shorter citation half-lives.

### Focused Analysis of Contemporary Articles (2010–2018)

A subanalysis of the 12 contemporary articles ( Table 3 ) revealed a lower mean total citation count (194.6 ± 65.5) compared with pre-2010 articles (259.8 ± 219.2) but a nearly twofold higher mean annual citation rate (16.32 vs. 8.87). Keyword analysis showed increased representation of treatment outcomes, postoperative care, risk assessment, and lymphedema, while keywords dominant in older articles, case reports, mandible reconstruction, and bone graft, were absent from the contemporary cohort. The predominant themes were free flap outcomes and complication analysis ( *n* = 4), monitoring and perioperative care optimization ( *n* = 3), lymphedema microsurgery ( *n* = 2), donor site morbidity assessment ( *n* = 2), and prognostic factor analysis ( *n* = 1). Notably, lymphedema microsurgery, entirely absent from the pre-2010 top 100, emerged as a new thematic cluster. The contemporary cohort demonstrated greater methodological diversity, including two prospective cohort studies, one systematic meta-analysis, and one consensus guideline. Geographically, while the United States remained the leading contributor ( *n* = 5), articles originated from eight countries, with Dort J.C.'s guidelines reflecting multinational collaboration across four nations.

**Table 3:** Characteristics of contemporary articles (2010–2018) in the top 100

Rank	First author	Year	Theme	Study design	Annual citation rate
15	Dort J.C.	2017	Perioperative care guidelines	Consensus guidelines	45.88
28	Wong C-H.	2010	Flap outcomes/monitoring	Review	17.93
40	Campisi C.	2010	Lymphedema microsurgery	Case series	13.47
41	Smit J.M.	2010	Free flap monitoring	Literature review	13.47
42	Hanasono M.M.	2010	Donor site morbidity	Prospective cohort	13.27
46	Basta M.N.	2014	Lymphedema treatment	Systematic review/meta-analysis	16.82
55	Patel R.S.	2010	Complication analysis	Retrospective cohort	11.47
68	Cho E.H.	2018	Comparative flap outcomes	Multicenter retrospective	22.86
83	Moreno M.A.	2010	Comparative reconstruction	Retrospective review	9.80
84	Kruse A.L.D.	2010	Prognostic factors	Narrative review	9.80
89	Pohlenz P.	2012	Flap outcomes	Retrospective cohort	11.08
97	Momoh A.O.	2011	Donor-site morbidity	Prospective cohort	10.07

## Discussion

This bibliometric analysis provides an overview of the most cited articles in reconstructive microsurgery, highlighting publication trends, influential authors, research focus areas, and citation impact across five decades. The highest output was observed in the 2000s, corresponding with significant advancements in free flap surgery. 21 22 The prominence of head and neck reconstruction (49.5%) aligns with previous bibliometric analyses demonstrating the high clinical demand and complexity associated with this subspecialty. 17 The consistent contributions of Hidalgo D.A., Wei F-C., and Taylor G.I. underscore the enduring impact of foundational studies in free flap design and reconstructive techniques. 21 23 24 The United States led in total contributions (49%), with significant output from Japan, Canada, Taiwan, and Australia, reinforcing previous reports of U.S. leadership in microsurgical research. 17 Institutionally, MD Anderson Cancer Center, Memorial Sloan-Kettering Cancer Center, and Mount Sinai Medical Center were the most represented, indicating that high-volume academic centers continue to drive innovation. The predominance of *Plastic and Reconstructive Surgery* (54% of articles) confirms its role as the primary platform for high-impact research in the field. 17

While the absence of Level I evidence, the predominance of U.S. contributions, and the concentration of influential studies in head and neck reconstruction are important findings, they are largely consistent with the established structure of the reconstructive microsurgery literature. The more important insight offered by this analysis is that the basis of influence appears to be changing. In the contemporary era, the most influential articles are increasingly those centered on evidence synthesis, consensus formation, and perioperative standardization rather than on technical description alone. The relative resurgence of Level V evidence in the 2010s reflects this shift and is driven primarily by reviews and consensus-based guidance rather than by low-quality primary research. The appearance of lymphedema microsurgery as a distinct thematic cluster further supports the view that the field is entering a new phase of development. That the highest annual citation rate in the dataset belonged to the consensus recommendations of Dort et al, rather than to a landmark technical article, reinforces the impression that influence in modern reconstructive microsurgery is increasingly tied to standardization, multidisciplinary care pathways, and broadly implementable clinical frameworks.

These findings indicate that reconstructive microsurgery has entered a more mature phase in which influential publications increasingly shape how care is organized, evaluated, and standardized across institutions. Earlier landmark studies established technical feasibility, donor site anatomy, and operative innovation. By contrast, more recent highly cited work has focused on evidence synthesis, perioperative pathways, complication reduction, and comparative outcomes. This shift likely reflects both methodological maturation and the growing importance of multidisciplinary care models in complex reconstruction.

The reliance on case series (19%) and retrospective cohort studies (12%), combined with the complete absence of Level I evidence and limited Level II representation (7.22%), underscores the urgent need for high-quality randomized controlled trials, multicenter prospective registries, and systematic reviews with meta-analyses. This mirrors findings in previous bibliometric analyses of plastic surgery research. 12 13 14 15 16 17

### Evolution of Reconstructive Microsurgery: An Integrative Analysis

Synthesizing the bibliometric, thematic, and evidence-level data, reconstructive microsurgery progressed through four overlapping phases. The technical feasibility phase (1970s–1980s) was characterized by pioneering anatomical studies and proof-of-concept case reports. Level IV evidence predominated appropriately, as the primary question was whether free tissue transfer could be safely performed. The contributions of Taylor, Harii, and Song established the anatomical foundation for all subsequent advances, and their sustained citation rates (Taylor G.I., 1975: 26.26/year after 50 years) reflect continued relevance as conceptual cornerstones. The clinical refinement phase (late 1980s–1990s) shifted the question from “can we do this?” to “how well can we do this?” Hidalgo's fibula free flap (1989) exemplifies this transition, identifying an optimal donor site for mandibular reconstruction with systematic evidence of its superiority. The emergence of large case series (>100 patients) and systematic complication analyses reflected the field's growing capacity for self-evaluation. The geographic concentration in the United States and a small number of high-volume centers underscore that clinical maturation was driven by institutional experience at major academic centers. The evidence-based optimization phase (2000s–2010s) introduced multi-institutional data aggregation, monitoring technology assessment, and the expansion into lymphatic surgery and perioperative care optimization. The progressive increase in annual citation rate from 6.87 (1970s) to 16.32 (2010s) reflects not only increased digital accessibility but also growing global engagement, as evidenced by the increasing number of contributing countries in later decades.

The incomplete transition to higher evidence levels is perhaps the most critical finding. The persistent absence of Level I evidence and the plateau of Level II contributions at fewer than 10% suggest structural barriers, including the ethical challenges of randomizing surgical techniques, heterogeneity of reconstructive defects, and small patient volumes at individual centers. Overcoming these barriers will require multicenter collaborative networks, standardized outcome reporting frameworks, and innovative designs such as pragmatic trials and prospective registries. The thematic trajectory also reveals important gaps. While head and neck reconstruction dominated (49.5%), emerging areas such as lymphatic microsurgery, vascularized composite allotransplantation, and robotic-assisted and artificial intelligence (AI)-guided microsurgery were underrepresented, largely because transformative work in these areas postdates the citation window. The observation that Dort J.C.'s 2017 ERAS consensus guidelines achieved the highest annual citation rate (45.88/year) suggests a growing appetite for standardized, multidisciplinary clinical protocols that may define the next generation of highly cited literature.

### Emerging Directions beyond the Citation Window

The broader recent literature suggests several transformative directions reshaping the field. Supermicrosurgery, surgery on structures less than 0.8 mm in diameter, has emerged as a rapidly evolving frontier, particularly in lymphatic reconstruction. 25 Dedicated robotic microsurgical platforms, including the Symani Surgical System and MUSA (Microsure, Eindhoven, the Netherlands), have enabled robot-assisted supermicrosurgical anastomoses with early studies demonstrating feasibility and promising outcomes. 26 27 Additionally, AI applications in flap monitoring, ERAS protocols adapted for microsurgical reconstruction, and ultrahigh-frequency ultrasound for preoperative mapping represent areas of active investigation. 25 Future bibliometric analyses should employ complementary metrics, such as citation velocity and Altmetrics, to capture these emerging contributions.

### Practical Implications and a Data-Driven Research Agenda

The absence of Level I evidence among the 100 most cited articles underscores an urgent need for higher-quality research. Despite inherent challenges, ethical constraints, surgical heterogeneity, and small patient populations, the field must prioritize multicenter prospective registries, comparative effectiveness studies, and well-designed systematic reviews targeting specific clinical questions amenable to higher-level designs.

The geographic concentration of nearly half of the highly cited studies in the United States highlights underrepresentation from regions with high clinical volumes, including East Asia, South America, and the Middle East. International collaborative networks and equitable access to high-impact publication platforms could address this imbalance. Similarly, emerging areas such as lymphatic microsurgery, vascularized composite allotransplantation, and technology-assisted microsurgery were underrepresented and warrant prioritization. Non-open-access articles had a higher mean but lower median citations than open-access articles, suggesting more consistent citation performance with open-access availability; funding agencies should consider supporting publication fees, particularly for lower-resource settings. Additionally, journals should enforce standardized funding disclosure, given that over three-quarters of top-cited studies did not report funding sources.

The emergence of lymphedema microsurgery as a distinct cluster in the contemporary cohort is also notable. Its absence from the pre-2010 top-cited literature and subsequent appearance among recent highly cited articles suggest a meaningful broadening of the field beyond traditional free flap reconstruction. This may reflect increasing clinical demand, improved diagnostic and imaging capabilities, and the expansion of supermicrosurgical techniques into lymphatic reconstruction.

Finally, the doubling of annual citation rates from the 1970s to the 2010s reflects accelerating knowledge turnover, while the sustained citation performance of foundational articles by Hidalgo, Taylor, and Song demonstrates that an enduring influence is achieved through contributions introducing novel conceptual frameworks or transformative techniques. Future investigators should target specific clinical questions amenable to higher-level designs, such as comparing reconstructive modalities through prospective multicenter protocols. 28

### Limitations and Future Recommendations

This study has several limitations. First, restricting inclusion to English-language publications may have excluded influential work published in other languages. Second, citation–age bias is inherent in any bibliometric analysis based on cumulative counts, as older articles have simply had more time to accrue citations. We attempted to address this by calculating annual citation rates and stratifying by decade and citation maturity, which confirmed that newer articles reach citation milestones more rapidly (Spearman ρ = −0.610, *p* < 0.001). That said, this adjustment assumes a relatively uniform citation trajectory and does not account for phenomena such as early citation bursts or late-stage decay. Future studies may benefit from field-weighted citation impact (FWCI), Altmetrics, and citation half-life analysis for more nuanced time-adjusted comparisons.

Third, the search strategy did not include “perforator flap” as a dedicated term. Although the existing keywords captured many perforator flap-related articles, certain landmark studies could not be retrieved through any keyword-based approach. Most notably, Koshima and Soeda's 1989 description of inferior epigastric artery skin flaps without rectus abdominis muscle (1,121 Scopus citations), 29 widely regarded as the foundational paper introducing the perforator flap concept, was missed because the original publication used purely anatomical terminology that predates the now-standardized nomenclature. This illustrates a well-recognized limitation of keyword-dependent bibliometric searches: Pioneering studies that introduced new concepts using descriptive rather than categorical language may escape retrieval even with comprehensive strategies. Supplementing keyword searches with author-based or citation-tracking approaches in future analyses would help capture such foundational contributions.

Fourth, citation count alone is an imperfect proxy for scientific quality; a highly cited paper may owe its citation volume to historical significance or conceptual novelty rather than methodological rigor. 27 Self-citation patterns and citation networks may also inflate counts for certain articles. Fifth, the most recent article in our dataset was published in 2018, which means that important post-2018 developments, including supermicrosurgery, robotic platforms, AI-assisted monitoring, and vascularized composite allotransplantation, fall outside the citation window. Future analyses should extend the timeframe and incorporate complementary metrics such as Altmetrics and citation velocity indices to capture emerging work.

Finally, we relied on Scopus as the sole citation database. While Scopus provides broad journal coverage and well-established bibliometric functionality, single-database reliance carries the risk of missing influential articles indexed exclusively in Web of Science or PubMed. Coverage differences are particularly relevant for older literature and journals with inconsistent historical indexing, and the composition and rank order of the top 100 may vary somewhat across databases. 30 A multidatabase approach integrating Scopus, Web of Science, and PubMed would improve completeness and allow cross-validation of citation metrics in future work.

Despite these limitations, this study offers a detailed overview of the most influential research in reconstructive microsurgery and provides a foundation for future investigation.

### Conclusion

This bibliometric analysis of the top 100 most cited Scopus-indexed articles in reconstructive microsurgery shows a shift from foundational anatomical and technical studies toward outcomes-focused research, evidence synthesis, and multidisciplinary standardization. Although major evidence gaps remain, including the persistent absence of Level I studies, the stronger contemporary signal is a change in what the field now cites most heavily: not only operative innovation, but also consensus-building, perioperative optimization, and broadly implementable frameworks of care. The emergence of lymphedema microsurgery and the citation prominence of guideline-based work suggest that the field is entering a more integrated and methodologically mature phase. These findings, interpreted within the scope of the Scopus-indexed literature retrieved by the applied search strategy, provide a basis for future research priorities centered on multicenter collaboration, standardized outcome reporting, and higher-quality comparative study designs.
